# Clarifying the Connection Between Parental and Child Stress: The Role of Coping Strategies and Parenting Behavior

**DOI:** 10.3390/children13101344

**Published:** 2026-10-03

**Authors:** Zhamilia N. Dzhansaidova, Dmitriy S. Kornienko, Natalia A. Rudnova

**Affiliations:** Federal Scientific Center for Psychological and Interdisciplinary Research, 125009 Moscow, Russia; zhamilyadzhansaidova@yandex.ru (Z.N.D.); rudnova.na@yandex.ru (N.A.R.)

**Keywords:** parental stress, child stress, coping, parenting, preschoolers

## Abstract

**Highlights:**

**What are the main findings?**

- Parental stress was positively associated with avoidance coping and negatively associated with positive reframing. Avoidance coping, in turn, predicted more punitive and inconsistent parenting, while positive reframing predicted less punishment and inconsistent discipline. Child stress was positively associated with punishment and inconsistent discipline.
- In a single structural model testing all pathways simultaneously, only two specific indirect pathways were statistically supported: parental stress–avoidance–punishment–child stress and parental stress–positive reframing–punishment–child stress. Most of the parental–child stress association was not explained by these coping–parenting mechanisms.

**What are the implications of the main findings?**

- Screening parents for avoidant coping and offering coping skills training—particularly techniques that reduce avoidance and foster positive reframing—could help prevent dysfunctional parenting and buffer children from the negative effects of parental stress, pending confirmation in future research.
- Routine psychological screening for both parents and children in educational settings (e.g., kindergartens, schools) may be a potential avenue for identifying at-risk families early, although this implication requires confirmation through longitudinal or intervention research before informing practice.

**Abstract:**

Background/Objectives. The association between child stress and parental stress has been well studied; however, the factors modifying this relationship have not been sufficiently examined. The current study investigates the roles of coping and parenting behavior in the relationship between parental and child stress based on a conceptual framework that unites different parental characteristics in explaining child stress. Methods. The study involved 271 mothers of preschoolers, who completed the following questionnaires: the Perceived Stress Scale, the Perceived Stress Scale for Children, the Alabama Parenting Questionnaire, and the Coping Orientation to Problems Experienced Questionnaire. Results. Parental stress is related positively to avoidant coping and negatively to positive reframing coping strategies. Different coping strategies, in turn, have significant associations with parenting behavior. Positive reframing relates negatively to punishment and inconsistent discipline, while avoidance has positive associations with ineffective parenting strategies, which in turn are linked with higher child stress. Punishment and inconsistent discipline coupled together are significantly and positively related to child stress. Conclusions. The current study clarified the association between parental and child stress, exploring the role of coping and parenting behavior. Positive reframing was associated with more favorable parenting and child-stress outcomes, while avoidant coping and dysfunctional parenting behavior were related to higher child stress. The findings demonstrate the importance of targeted interventions that can help mitigate the negative effects of stress on children and improve overall family well-being.

## 1. Introduction

Stress is one of the most studied phenomena and could be defined as individual response to a real or imagined stressor [1]. During childhood the ability to respond to an actual stressor is tied to the level of children’s physical and mental development. As a result, children of different ages exhibit varying outcomes of experiencing stress [2]. Stress in infancy can disrupt physiological rhythms; during preschool age it can cause regression of developed skills such as thumb sucking, fears of parting with parents, and enuresis, as well as somatic symptoms (abdominal pain, for example); and at school age, stress can foster difficulties with memorization and concentration, resulting in a decline in academic performance [3,4,5]. Severe or chronic stress in children results in the most devastating and enduring consequences, increasing the likelihood of emotional and behavioral issues, as well as the onset and persistence of mental and somatic disorders [6].

Among the many factors that influence child well-being and stress levels, parental factors play a crucial role [7,8,9,10]. Parents serve as the primary caregivers for their children, fulfilling all their physiological and psychological needs during early stages of development. Previous studies confirmed that parental stress and child stress are mutually related and interdependent [11,12,13], and parental stress has a broader impact on children’s well-being [14,15]. In this study, parental stress refers to parents’ general perceived stress across domains of daily life, rather than stress arising specifically from the parenting role itself. The two are conceptually related but not interchangeable, and this distinction is maintained throughout the manuscript.

To clarify the mechanisms linking parental and child stress, prior research has examined potential mediators of this association [16,17]. Parenting has been identified as one of potential mediators [18]. According to previous research, increased parental stress can influence the deterioration of various aspects of parenting [19,20]. Parents who experience severe stress are more likely to abuse their children, show less warmth towards them, spend less time with their children, be less involved in games and other activities [21] and have relationships that are less close and more conflictual [16]. Parents exposed to severe stressors also pay less attention to regulating their children’s behavior and emotions [22] and use harsher disciplinary practices [23].

Parenting behavior refers to parents’ observable disciplinary and caregiving practices, operationalized here via positive parenting, punishment, and inconsistent discipline. Deterioration of parental practices, in turn, has been linked to several detrimental consequences for a child, including decreased psychological well-being and increased stress levels [24]. For example, an authoritarian parenting style is associated with behavioral difficulties; excessive parental control is linked to anxiety and irritation in children; the use of both physical and non-physical punishments is associated with a variety of psychological problems in children [25,26,27,28]. Based on previous studies, it can be assumed that parenting behavior may be a factor explaining the association between parental and child’s stress. Increased parental stress may be linked to reduced effectiveness of parental behavior, which may be associated with elevated child stress levels.

Nevertheless, the “parental stress → parenting behavior → child stress” model requires further improvement. First, empirical research has raised doubts about the presence of direct linear relationships between these components [29]. Second, relying on this model would limit practical recommendations for parents to the assertion that learning positive parenting practices is important for avoiding negative effects on the child during periods of heightened stress. However, such an approach may put additional pressure on parents already experiencing stress. Therefore, further research is needed to identify components which can help to support parents in preventing worsening parenting practices and, in turn, affecting the child.

In Abidin’s parenting stress model, such components are referred to as resources, and one of the most important resources is coping strategies, which may serve a buffering role [29]. Two arguments support the inclusion of coping in the “parental stress → parenting behavior → child stress” model. First, researchers have suggested that parenting behavior changes less as a direct consequence of stress itself than because of ineffective coping with that stress [30,31]. Second, coping strategies are potentially modifiable factors which can be changed through psychological interventions [32]. This provides a way to support parents without adding further pressure.

Coping is defined as cognitive, emotional, and behavioral efforts aimed at preventing threats and reducing stress [33]. Maladaptive coping can lead to various consequences, including changes in parenting behavior, e.g., parents’ lack of adaptive coping mechanisms significantly predicted child abuse during the COVID-19 pandemic [31]. It has also been found that parents who use avoidant coping often do not seek to change the source of stress when faced with stress, and therefore may, for example, have problems correcting their parenting strategies if necessary [30]. Problem-oriented coping is an adaptive resource for positive and supportive parenting, while emotion-oriented coping contributes to negative parenting styles [34,35]. For instance, problem-oriented coping involves actively planning and taking action to address the source of stress, whereas emotion-oriented coping involves expressing or discussing one’s feelings about the stressor rather than addressing it directly. However, most of previous studies identify only general patterns between inadequate stress coping and worsened parenting strategies, without specifying relationships between individual coping strategies and specific parenting behaviors [31]. Researchers also note pronounced heterogeneity in the described findings [36]. In this regard, the relationship between parental coping and both parenting behavior and parental and child stress requires further investigation.

Previous studies have typically examined the relationships between parental stress and coping [37], between parental stress and changes in parenting behavior [34,38], and between parenting behavior and child outcomes [27,28]. Nevertheless, it seems important to consider all four components in their interrelation, as this could not only increase our knowledge about the parent–child stress relationship, but it could also help in the creation of interventions to reduce stressors for families and improve the health of both parents and children. A recent study proposed a promising conceptual framework that unites all four components into a single model based on Abidin’s theoretical framework [29,35]. The suggested model, in Figure 1, illustrates the link between parental stress and child stress via parental coping strategies and parenting styles. The pathway of associations can be described as follows: parental coping serves as a mediator between parental stress and parenting styles, whereas parenting styles mediate the effect of parental coping on children’s behavioral problems.

The model was successfully tested on a sample of 200 South Korean mothers and daughters aged from 8 to 14 years. It was found that parental stress (caused by family and personal roles) is associated with emotion-focused coping; this coping mechanism, in turn, increases negative parenting behaviors (emotional rejection, chaotic fostering, and coercion as the main children’s behavior-regulating tactic), which subsequently have a harmful impact on children’s stress levels and their internalizing and externalizing problem behaviors [35].

The purpose of the current study is to clarify the relationship between parental stress, coping, parenting behavior, and child stress based on the model proposed by Cho and colleagues [35]. The results of the theoretical analysis and earlier studies suggest the following hypotheses:

(1) Parental stress is positively associated with the use of maladaptive coping strategies and negatively associated with the use of adaptive coping strategies.
(2) Maladaptive coping strategies are positively associated with dysfunctional parenting behavior and negatively associated with positive parenting.
(3) Dysfunctional parenting behavior is positively associated with child stress.
(4) Parental stress is associated with child stress through a sequential pathway involving coping and parenting behavior, such that coping strategies statistically mediate the association between parental stress and parenting behavior, and parenting behavior in turn mediates the association between parental coping and child stress.

To further develop Cho’s model, this study was conducted with several enhancements. In contrast to Cho’s study, our research focuses on children of middle preschool age, in which the influence of parental factors may differ from that observed in primary-school-age children and early teenagers. Second, the present study was conducted on a non-clinical sample, while Cho’s model was previously tested on children diagnosed with precocious puberty undergoing hospital treatment during the study. Third, this study is conducted in a different cultural context: Cho’s model was tested in South Korea, whereas the respondents to this study are residents of Central Russia. Fourth, this study extends our team’s earlier work [14], which proposed that maternal stress depletes the resources needed for positive parenting. That previous study examined the impact of maternal stress and parenting practices as predictors of preschoolers’ stress but did not address coping strategies. The present study incorporates coping as an explicit mediating mechanism between parental stress and parenting behavior, providing a direct empirical test of the pathway that the earlier study [14] could only speculate about.

## 2. Materials and Methods

### 2.1. Participants

The study involved 279 parents of preschool children, among them were 271 mothers which formed the study sample. Because fathers accounted for a small proportion of the initial sample (only 8 respondents) that is too few to support separate analysis or to model respondent gender as a covariate, only mothers’ responses were retained for the analyses reported here to preserve a homogeneous, single-informant sample. Mothers are aged 24 to 50 years (*M* = 37.3, *SD* = 4.7), 85.4% of mothers were married, 87.6% had higher education. All participants lived in the urban areas. Children attended preschool educational institutions, the age of children was from 54 to 83 months (*M* = 65.5, *SD* = 4.5), 54% were girls.

### 2.2. Procedure

This study was conducted in 2025. The involvement of parents was facilitated by the administrations of preschool organizations who provided the link to the questionnaires to parents. Participants were recruited from five educational institutions in Moscow, whose administrations distributed the survey link to parents of children enrolled in the relevant preschool groups. Because the survey was distributed by institution administrators rather than to individually tracked recipients, the exact number of parents invited and the resulting response rate could not be determined. Eligible participants were parents aged 18 years or older, of any marital status, with at least a secondary education, and either employed or engaged in homemaking, across a range of family socioeconomic backgrounds. A total of 279 parents completed the survey. Data were collected using online forms, and all materials were presented in the Russian language. The confidential survey was completed voluntarily, with written informed consent obtained from all respondents. Researchers implemented data anonymization procedures to ensure participant confidentiality. The research procedure adhered to the ethical guidelines established by the Russian Psychological Society.

### 2.3. Measures

To assess the parental stress, a short form of the Perceived Stress Scale (PSS-4) was used, developed by Cohen and adapted to Russian by Zolotareva [39,40]. The tool consists of 4 items, each of which parents had to evaluate on a four-point Likert scale (where 1 = never, and 4 = almost all the time). Cronbach’s alpha for PSS was 0.7. PSS-4 measures general perceived stress rather than stress specific to parenting demands; it was selected because Abidin’s model treats parental resources broadly, such that general stress—not only parenting-specific stress—can affect parenting behavior [29].

To assess the child stress, the Perceived Stress Scale for Children (PSS-C), developed by White, was used [41]. The Russian-language version of PSS-C has a two-factor structure that includes factors of Distress and Well-being. The version of the scale used in the study was adapted for evaluation by parents [15]. In this study, only a subscale of Distress was used, which included 6 questions about the condition of children presented to the parent. Parents were asked to evaluate each item on a four-point scale (where 1 = never, and 4 = almost all the time). Cronbach’s alpha for PSS-C was 0.7. The Distress subscale was selected because it most directly captures children’s affective and physiological reactions to stress, in line with the study’s focus on stress reactivity, whereas the Well-being subscale reflects a broader and more stable disposition that was not the focus of this research. Accordingly, the child-stress findings reported here should be interpreted as reflecting the Distress dimension specifically, rather than children’s overall psychological well-being.

To identify the features of parenting behavior, the Alabama Parenting Questionnaire, Preschool Revision (APQ–PR) was used, developed by Clerkin and adapted to the Russian language by Loginova [42,43]. The questionnaire consists of 17 items that make up 3 scales: positive parenting (Cronbach’s alpha = 0.8), inconsistent discipline (Cronbach’s alpha = 0.7) and punishment (Cronbach’s alpha = 0.6). This questionnaire was also filled out by parents who were asked to assess their agreement with various statements about the specifics of their behavior (where 1 = never, 5 = always).

The short version of the Coping Orientation to Problems Experienced questionnaire (COPE-30) which was developed based on the full version by Carver was used to evaluate parents’ coping strategies [33,44]. The full questionnaire includes 30 items and allows for identification of 15 stress-management strategies. The short version was limited to 15 items (1 point for each coping strategy). Despite the reliability of the questionnaire, there are concerns about the secondary structure, which may vary depending on the sample and other conditions. The preliminary analysis was conducted with the goal of identifying the elements that accurately reflect the coping mechanisms relevant to the population being studied. The result of exploratory factor analysis resulted in the following second-order factor structure: emotionally focused coping (the emotions expression, the seeking of emotional and social support), problem-focused coping (planning, suppression of other activities, acceptance, and restraint) avoidance (behavioral and mental disengagement, denial, and disorganization) and positive reframing (only the positive reframing scale was included). Sampling adequacy for this exploratory factor analysis was acceptable (KMO = 0.633; Bartlett’s test of sphericity: χ^2^(55) = 439.30, *p* < 0.001), and the four-factor solution showed good fit to the data (χ^2^(17) = 20.09, *p* = 0.270; CFI = 0.992; TLI = 0.974; SRMR = 0.022; RMSEA = 0.026, 90% CI [0, 0.064]). The Cronbach’s alphas for the scales based on the second-order factor vary from 0.5 to 0.7 which is questionable, but those reliability coefficients could be accepted for the short versions of questionnaires [45,46].

### 2.4. Statistical Analysis

The Jasp 0.19.3 software was used for statistical analysis. Spearman correlation analysis was employed to find the links between the study variables. A series of regression analyses was conducted to determine the impact on the parental features to the child’s stress variable. A mediation analysis using bootstrapping (1000 resamples, bias-corrected 95% confidence intervals) was conducted to test the hypothesized mediation pathways. In the preliminary analysis, the Shapiro–Wilk test was made to assess the data distribution. All hypothesized direct and indirect paths were estimated simultaneously within a single pre-specified structural equation model (R package lavaan 0.6-17), and each specific indirect effect was considered statistically supported only if its bias-corrected (BCa) bootstrap 95% confidence interval excluded zero. The test revealed a violation of normality, which led to the use of nonparametric methods.

## 3. Results

### 3.1. Common Method Bias Test

The Harman single-factor test was used to assess common method bias [47]. The first unrotated factor accounted for 12.7% of the total variance, which is below the critical value of 40%. This indicates that common method bias does not pose a significant threat to the validity of research findings.

### 3.2. Descriptive Statistics and the Associations Between Study Variables

Descriptive statistics present the overall expression of the study variables. Table 1 demonstrates that mothers assess their children’s stress level higher than their own stress. Among parenting behavior, positive parenting expression is the highest, while punishment and inconsistent discipline are less expressed. All coping strategies are presented in z-scores and demonstrate moderate level. According to Spearman’s correlation analysis (Table 1), parental stress is associated with child stress, and the following coping strategies: positively with avoidance and negatively with positive reframing. Parent’s maladaptive coping strategies have found significant associations with negative parenting behavior. Avoidance showed a positive association while positive reframing demonstrated negative association with punishment and inconsistent discipline as parenting practices. At the same time positive parenting had a positive association with positive reframing, problem-focused and emotion-focused coping, and negative with avoidance. The inconsistency of parenting behavior and the use of punishment is positively related to child stress which support the third hypothesis. In addition, child stress is linked with parental avoidance. Child gender, child age, and maternal age were also examined as potential covariates; none of the three showed a significant correlation with any of the study variables (Table 1), and they were therefore not included in the structural model reported in Section 3.4.

### 3.3. The Impact of Parental Characteristics on a Child Stress

With the goal to investigate (1) the connection between parental stress and coping strategies, (2) coping strategies and parenting strategies, and (3) parenting strategies and child stress, we replicated the statistical procedure and conducted a series of regression analyses, according to the study conducted by Cho and colleagues [35].

In the first stage, regression models were built with coping strategies as the dependent variables and parental stress as the predictor (Table 2). Parental stress was a significant positive predictor of avoidance (*β* = 0.36, *p* < 0.001) and a significant negative predictor for positive reframing (*β* = −0.28, *p* < 0.001), while parental stress was not a significant predictor for either emotion-focused or problem-focused coping.

In the second stage, models were constructed where parenting strategies served as the dependent variables and coping strategies as the predictors (Table 2). Positive parenting as significant predictors had emotion-focused coping (*β* = 0.15, *p* < 0.05), problem-focused coping (*β* = 0.16, *p* < 0.01), and avoidance (*β* = −0.13, *p* < 0.05). Punishment was connected with positive reframing as its negative predictor (*β* = −0.15, *p* < 0.05) and avoidance as its positive predictor (*β* = 0.22, *p* < 0.001). Inconsistent discipline as significant predictors had emotion-focused coping (*β* = 0.13, *p* < 0.05), problem-focused coping (*β* = 0.12, *p* < 0.05), avoidance (*β* = 0.15, *p* < 0.05) and positive reframing (*β* = −0.13, *p* < 0.05).

In the third stage, a regression model was built for the child stress as the dependent variable and parenting strategies as predictors. It was discovered that punishment (*β* = 0.19, *p* < 0.01) and inconsistent discipline (*β* = 0.13, *p* < 0.05) were related positively to child stress, while positive parenting was not associated significantly with child stress (Table 2). Figure 2 shows the variables that revealed significant associations with parental or child stress as a result of regression analysis.

### 3.4. The Mediation Role of Coping Strategies and Parenting Styles

Consistent with hypothesis 4, we tested a single structural model specifying the complete hypothesized chain: parental stress—coping (emotion-focused, problem-focused, avoidance, positive reframing)—parenting behavior (positive parenting, punishment, inconsistent discipline)—child stress, together with a direct path from parental stress to child stress. All hypothesized paths were estimated simultaneously, without preselecting paths based on prior significance (full results for all twelve specific pathways are reported in Supplementary Table S1). The total association between parental stress and child stress (β = 0.306, 95% CI [0.201, 0.411]) was decomposed into a direct effect (β = 0.280, 95% CI [0.174, 0.385]) and a total indirect effect through the twelve tested pathways (β = 0.026, 95% CI [0.006, 0.046]) (Table 3). Of the twelve specific pathways tested, only two showed bootstrapped 95% CIs excluding zero: parental stress–avoidance–punishment–child stress (β = 0.013; unstandardized bootstrapped 95% CI [0.003, 0.038]) and parental stress–positive reframing–punishment–child stress (β = 0.007; unstandardized bootstrapped 95% CI [0.001, 0.019]). The remaining specific pathways, including those via inconsistent discipline and via emotion- or problem-focused coping, were not statistically supported (Supplementary Table S1).

## 4. Discussion

The objective of this study was to investigate factors that may elucidate the relationship between parental stress and child stress and the features that bind them. Based on Abidin’s parental stress model and the narrower conceptual framework proposed by Cho and colleagues, it was hypothesized that parental stress and child stress are linked through coping strategies and parenting strategies [29,35]. Because parental stress, coping, parenting behavior, and child stress were all assessed through maternal self-report, shared method variance may have inflated some of the observed associations; although a Harman’s single-factor test suggested that this is unlikely to be the dominant explanation, the findings below should be interpreted with this possibility in mind.

The findings of the study indicate that parental stress is positively associated with avoidance and negatively associated with positive reframing. These results support the first hypothesis, indicating that as parents experience more stress, they are more likely to avoid positive and effective stress management. It corroborates with the notion that high levels of stress can impede the use of adaptive coping strategies [30,48]. A systematic review has shown that parents of children with autism, compared to parents of typically developing children, are more likely to use avoidance and less likely to seek social support, which may be related to the unavailability of adaptive strategies in the context of chronic stress [49]. The absence of the association between the parental stress and emotion-focused or problem-focused coping strategies could be explained by the influence of the other situational factors, like the source of stress, or individual factors like parent’s personality features. As an example, in Cho’s research it was identified that emotion-focused or problem-focused coping strategies were correlated with distinct types of stressors [35].

It is also important to note that previous research has often considered coping strategies as predictors of parental stress, asking what reduces or buffers stress in adults [48]. In contrast, our study utilizes a distinct conceptual framework to explore how different coping strategies of parents may be connected with their behaviors toward children during stressful conditions, thereby connecting parental stress to child outcomes. This approach may complement previous research by addressing a different perspective of the stress process.

Considering coping strategies as predictors of parenting styles is consistent with Belsky’s process model, in which parental personal characteristics and resources are among the most important determinants of parenting [50]. Regarding the coping strategies as factors for parenting, the current study found that avoidance emerged as a negative factor for parenting, while positive reframing emerged as a positive factor, which corroborates with previous findings [30]. Parents inclined to minimize contact with the stressor, engage in denial, or retreat into fantasy are more prone to inconsistent discipline and less inclined toward positive parenting practices. This may be because avoidance does not solve the problem but may only temporarily reduce the impact of the stressor, whose effects are still felt by the parent over time and can lead to inconsistent parental behavior. In contrast, positive reframing may help parents reinterpret stressful situations in a more constructive way, reducing the emotional charge of the stressor and freeing cognitive resources for parenting.

Generalizing, the obtained results about the associations between parental stress-management features and parenting behavior support the second hypothesis and are mostly in line with previous research that indicated that increased parental stress can lead to a deterioration in various parenting practices [23], while adequate stress coping may serve a buffering function in this relationship [30].

The current study shows that dysfunctional parenting behavior tactics, the use of punishment and inconsistent discipline is associated with high child stress which is in line with most previously reported findings. These parenting strategies deprive the child of crucial elements of psychological well-being: safety, stability, and predictability, thereby leading to various negative short-term and long-term consequences [26,27]. However, it is important to note that cultural context plays an important role in understanding parenting practices. The cultural normativeness hypothesis suggests that associations between parenting and child outcomes may differ in cultural contexts where particular practices are more normative and accepted [51]. Previous findings indicate that the meaning, acceptability, and consequences of disciplinary practices are shaped by cultural norms, which may influence the interpretation and generalizability of the present results [52]. Generalizability is limited by the sample composition: the participating mothers were all urban residents with above-average educational attainment, as 87.6% held higher education degrees. Future cross-cultural studies could further clarify these contextual differences.

Combining the findings of the study, it is possible to conclude that parental stress is associated with a greater tendency to avoid stressful situations. Avoidance, as a maladaptive stress-management technique, is in turn linked with higher levels of punishment and inconsistent discipline, and with lower levels of positive parenting. Dysfunctional parenting characteristics, punishment coupled with inconsistent discipline, are associated with higher levels of child stress. These regression-level associations are broadly consistent with hypothesis 4, which the structural model reported below examines more directly. Although emotional and problem-focused coping contribute positively to parenting, the latter does not correlate with child stress, which may be considered the primary distinction from Cho and colleagues’ study [35]. Comparing the facts in the current study and Cho and colleagues’ study, it may be speculated that the relationship between parental and child stress modified by the maladaptive coping strategies and dysfunctional parenting is generally similar regardless of the age of child and place of residence. Nevertheless, the direct comparison could be made with caution, as the Korean study used a clinical sample.

Additionally, based on Cho and colleagues’ model, we tested whether coping and parenting behavior jointly account for the association between parental and child stress. The results are compatible with a modest, partial pathway linking parental and child stress through avoidance coping and punitive discipline, and, to a lesser extent, through positive reframing and punitive discipline. However, most of the association between parental and child stress was not accounted for by the coping–parenting mechanisms examined here, indicating that other, unmeasured factors likely play a larger role than the specific mechanism proposed in this model. Given the cross-sectional design, these findings should be interpreted as associations consistent with the proposed model rather than as evidence of causal or temporal transmission of stress from parent to child.

Several potential implications for intervention, which require further investigation rather than constituting direct practice recommendations, can be considered. First, implementing regular and available psychological screening for both children and parents, for example, in educational settings such as kindergartens and schools, could represent a potential step toward identifying at-risk families, pending evaluation in future research. Second, given that avoidance coping was associated with both heightened parental stress and maladaptive parenting behavior, parents reporting high levels of avoidance could potentially benefit from coping skills training. Such training programs might incorporate cognitive-behavioral therapy techniques, which have demonstrated efficacy in reducing avoidant behavior across multiple contexts [53]. Moreover, a core target of cognitive-behavioral therapy is the modification of maladaptive cognitions through cognitive restructuring. This strategy may be useful for teaching parents positive reframing, a coping strategy that showed a protective role in the parent–child stress link in the present study. Overall, these findings highlight the importance of attending not only to parent–child relationship quality but also to parents’ own psychological well-being, though these implications should be tested directly before informing practice.

Our study has several limits. First, the cross-sectional design precludes any causal inference, and the direction of effects cannot be established from our data; longitudinal or experimental studies are required to confirm the proposed mediation pathways. In addition, all models assumed linear relationships among the study variables; potential non-linear or reciprocal associations were not examined and cannot be ruled out. Second, all the data used in the study were obtained by parents, which may distort the results due to the influence of subjective factors. Subsequent research could implement alternative methods of a child’s stress level evaluation. Third, the study participants were only mothers, which does not allow us to consider the contribution of other significant adults to children. Future research can be improved by including more other caregivers in the sample, such as fathers. Fourth, despite revealed paths between parental stress, coping strategies, parenting behavior, and child stress, the models explain a modest percentage of the variance. This indicates child stress is affected by various factors, including socio-demographic variables and contextual components, such as the overall emotional climate within the family. Fifth, several measures have limitations. The PSS-4 assesses general perceived stress, which may not fully capture parenting-specific stressors. Similarly, only the Distress subscale of the PSS-C was used, narrowing the scope of child stress assessed. The short-form COPE further reduces measurement precision relative to the full inventory. In addition, some measures did not demonstrate satisfactory reliability. Because measurement error attenuates observed associations relative to their true magnitude, this may have led to underestimation of the corresponding paths, an effect likely compounded in the specific indirect pathways, which combine several such paths and may accordingly show wider, less stable confidence intervals—partly explaining why only two of the twelve specific indirect pathways reached statistical significance. These measurement constraints should be considered when interpreting the present findings.

## 5. Conclusions

The present study seeks to clarify the link between parental stress and child stress by examining the sequential roles of coping strategies and parenting behaviors. Generalizing, it can be concluded that parental stress is associated with behavioral and mental disengagement, as well as denial when confronted with stressful situations. This, in turn, is connected with the use of negative fostering techniques such as punishment and contradictory behavior, which further may increase stress in children. These findings are consistent with a model in which coping strategies and parenting behavior help account for the association between parental and child stress.

## Figures and Tables

**Figure 1:**
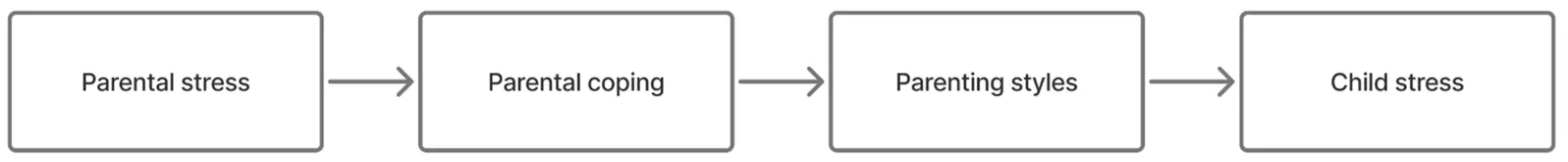
Conceptual framework of the relationship between parental and child stress.

**Figure 2:**
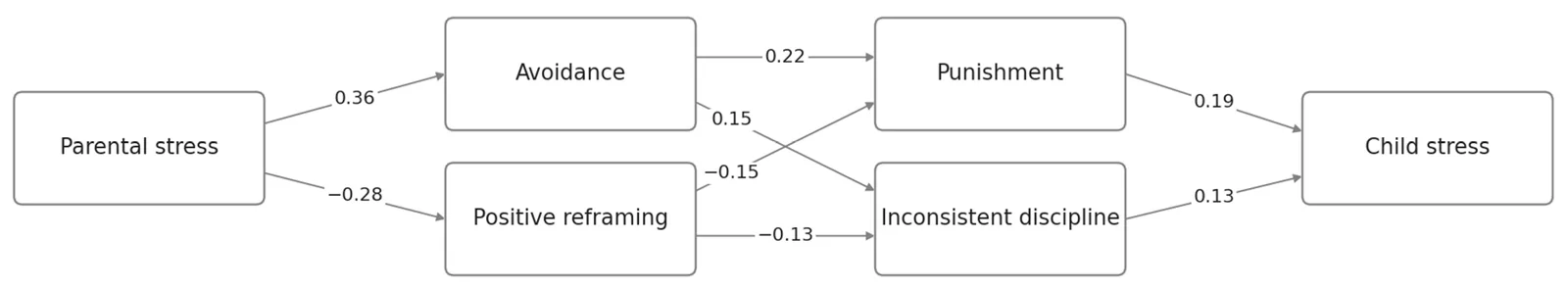
The model of the relationship between parental and child stress through coping strategies and parenting behavior. Note. Standardized coefficients (β) are depicted. The arrows indicate significant associations.

**Table 1:** Descriptive statistics and correlations of the variables.

Variable	M	SD	1	2	3	4	5	6	7	8	9	10	11	12
1. Child gender	1.545	0.499	—											
2. Child age (months)	65.515	4.532	0.010	—										
3. Parent age (years)	37.300	4.735	0.013	0.115	—									
4. Child stress	1.839	0.522	−0.067	0.032	−0.062	—								
5. Parental stress	0.944	0.647	0.024	0.113	−0.036	0.357 ***	—							
6. Positive parenting	4.507	0.429	0.005	0.009	−0.040	−0.058	−0.134 *	—						
7. Punishment	1.738	0.484	−0.104	−0.013	−0.106	0.267 ***	0.159 **	−0.142 *	—					
8. Inconsistent discipline	2.938	0.645	−0.056	0.114	−0.064	0.194 **	0.236 ***	0.080	0.200 **	—				
9. Emotion- focused coping	0.007	0.883	−0.029	0.078	−0.063	0.059	−0.010	0.184 **	0.012	0.160 **	—			
10. Problem- focused coping	3.815 × 10^−4^	0.830	−0.101	−0.105	0.013	−0.053	−0.029	0.174 **	0.103	0.114	0.117	—		
11. Avoidance	0.005	0.799	−0.058	0.062	−0.044	0.265 ***	0.362 ***	−0.125 *	0.246 ***	0.182 **	−0.012	−0.067	—	
12. Positive reframing	0.002	0.879	0.016	−0.108	0.050	−0.079	−0.280 ***	0.186 **	−0.164 **	−0.154 *	0.057	0.031	−0.016	—

Note. *N* = 271. *M* = mean, *SD* = standard deviation. Child gender: male = 0, female = 1. Child stress and Parental stress are mean item scores on the PSS-C (Distress subscale) and PSS-4, respectively (see Section 2.3). Positive parenting, Punishment, and Inconsistent discipline are mean item scores on the corresponding APQ-PR subscales. Emotion-focused coping, Problem-focused coping, Avoidance, and Positive reframing are standardized (z-score) factor scores from the exploratory factor analysis of the COPE inventory (*M* = 0, *SD* = 1 by construction); positive values indicate greater relative use of that strategy. * *p* < 0.05; ** *p* < 0.01; *** *p* < 0.001.

**Table 2:** The results of a series of regression analyses.

Predictor	Dependent Variable	b	SE	β	t	R2
1. Parental stress → Coping						
Parental stress	Emotion-focused coping	0.014	0.021	0.041	0.664	0.002
Parental stress	Problem-focused coping	0.001	0.020	0.004	0.068	0.000
Parental stress	Avoidance	0.110 ***	0.018	0.358 ***	6.154	0.128
Parental stress	Positive reframing	−0.094 ***	0.020	−0.277 ***	−4.634	0.077
2. Coping → Parenting behavior						
Emotion-focused coping	Positive parenting	0.073 *	0.029	0.149 *	2.477	0.085
Problem-focused coping		0.083 **	0.031	0.159 **	2.641	
Avoidance		−0.070 *	0.033	−0.130 *	−2.157	
Positive reframing		0.057	0.030	0.116	1.936	
Emotion-focused coping	Punishment	0.001	0.032	0.002	0.032	0.077
Problem-focused coping		0.053	0.034	0.095	1.560	
Avoidance		0.127 ***	0.036	0.217 ***	3.572	
Positive reframing		−0.078 *	0.032	−0.146 *	−2.417	
Emotion-focused coping	Inconsistent discipline	0.092 *	0.044	0.126 *	2.082	0.073
Problem-focused coping		0.094 *	0.047	0.121 *	1.997	
Avoidance		0.123 *	0.049	0.152 *	2.508	
Positive reframing		−0.098 *	0.044	−0.133 *	−2.197	
3. Parenting behavior → child stress						
Positive parenting	Child stress	−0.282	0.444	−0.039	−0.636	0.067
Punishment		1.288 **	0.411	0.193 **	3.132	
Inconsistent discipline		0.631 *	0.299	0.129 *	2.111	

Note. *b* = unstandardized regression coefficient; *SE* = standard error; β = standardized regression coefficient; *R*^2^ = variance explained by the corresponding regression model (block of predictors). * *p* < 0.05; ** *p* < 0.01, *** *p* < 0.001.

**Table 3:** The results of mediation analysis (Total, direct, and total indirect effects).

Effect	β	SE	95% CI	*p*
Total effect (Parental stress—Child stress)	0.306	0.054	[0.201, 0.411]	<0.001
Direct effect (controlling for all mediators)	0.280	0.054	[0.174, 0.385]	<0.001
Total indirect effect (sum of 12 pathways) ^a^	0.026	0.010	[0.006, 0.046]	0.012

Note: ^a^ The total indirect effect and its CI are reported here in standardized form for comparability with the other rows; the underlying bootstrap significance test uses the unstandardized effect (*b* = 0.031, *SE* = 0.013, 95% CI [0.010, 0.062], *p* = 0.015). Of the twelve specific indirect pathways tested (Supplementary Table S1), only two had bootstrapped 95% CIs excluding zero: parental stress–avoidance–punishment–child stress and parental stress–positive reframing–punishment–child stress.

## Data Availability

The data presented in this study are available from the corresponding author upon request.

## Supplementary Materials

The following supporting information can be downloaded at: https://www.mdpi.com/article/10.3390/children13101344/s1, Table S1: Specific indirect effects (parental stress—coping—parenting—child stress), full model.
