# Supplementary material for: Clarifying the Connection Between Parental and Child Stress: The Role of Coping Strategies and Parenting Behavior
Source: Children (Basel). 2026 Oct 3;13(10):1344. doi: 10.3390/children13101344 (PMC13703139; doi:10.3390/children13101344)
Supplement: Supplementary file 1 [file children-13-01344-s001.zip › children-4545534-supplementary.pdf]

Supplementary Table S1.

Specific indirect effects (parental stress —coping — parenting —child stress), full model.

All twelve pathways from the pre-specified structural model reported in Table 3 (main text), estimated simultaneously with no post hoc path selection. N = 271 (FIML); 1,000 bias-corrected (BCa) bootstrap resamples.

Parental coping	Parenting behavior	β	b	BCa 95% CI (b)	Significance
**Avoidance**	**Punishment**	**.013**	**.015**	**[.003, .038]**	**Yes** [a]
	Inconsistent discipline	.003	.004	[−.001, .020]	No
	Positive parenting	.000	.000	[−.005, .011]	No
**Positive reframing**	**Punishment**	**.007**	**.008**	**[.001, .019]**	**Yes** [a]
	Inconsistent discipline	.002	.003	[−.002, .012]	No
	Positive parenting	.000	.000	[−.004, .007]	No
**Emotion-focused coping**	Punishment	.000	.000	[−.001, .003]	No
	Inconsistent discipline	.000	.000	[.000, .006]	No
	Positive parenting	.000	.000	[−.003, .001]	No
**Problem-focused coping**	Punishment	.000	.000	[−.003, .005]	No
	Inconsistent discipline	.000	.000	[−.001, .003]	No
	Positive parenting	.000	.000	[−.002, .002]	No

[a] Significance is determined by whether the bias-corrected (BCa) bootstrap 95% CI for the unstandardized indirect effect excludes zero, following standard practice for mediation analysis (MacKinnon et al., 2004; Preacher & Hayes, 2008), rather than by a normal-theory (Wald) p-value — the sampling distribution of a product of coefficients is typically skewed, so a p-value from the bootstrap SE understates the precision of the BCa CI.
